# Small Target-YOLOv5: Enhancing the Algorithm for Small Object Detection in Drone Aerial Imagery Based on YOLOv5

**DOI:** 10.3390/s24010134

**Published:** 2023-12-26

**Authors:** Jiachen Zhou, Taoyong Su, Kewei Li, Jiyang Dai

**Affiliations:** 1 School of General Aviation, Nanchang Hangkong University, Nanchang 330063, China; 2204081200003@stu.nchu.edu.cn; 2 School of Information Engineering, Nanchang Hangkong University, Nanchang 330063, China; 36039@nchu.edu.cn (K.L.); djiyang@163.com (J.D.)

**Keywords:** drone aerial imagery, feature fusion network, receptive field feature extraction module, dynamic object detection head, small objects

## Abstract

Object detection in drone aerial imagery has been a consistent focal point of research. Aerial images present more intricate backgrounds, greater variation in object scale, and a higher occurrence of small objects compared to standard images. Consequently, conventional object detection algorithms are often unsuitable for direct application in drone scenarios. To address these challenges, this study proposes a drone object detection algorithm model based on YOLOv5, named SMT-YOLOv5 (Small Target-YOLOv5). The enhancement strategy involves improving the feature fusion network by incorporating detection layers and implementing a weighted bidirectional feature pyramid network. Additionally, the introduction of the Combine Attention and Receptive Fields Block (CARFB) receptive field feature extraction module and DyHead dynamic target detection head aims to broaden the receptive field, mitigate information loss, and enhance perceptual capabilities in spatial, scale, and task domains. Experimental validation on the VisDrone2021 dataset confirms a significant improvement in the target detection accuracy of SMT-YOLOv5. Each improvement strategy yields effective results, raising the average precision by 12.4 percentage points compared to the original method. Detection improvements for large, medium, and small targets increase by 6.9%, 9.5%, and 7.7%, respectively, compared to the original method. Similarly, applying the same improvement strategies to the low-complexity YOLOv8n results in SMT-YOLOv8n, which is comparable in complexity to SMT-YOLOv5s. The results indicate that, relative to SMT-YOLOv8n, SMT-YOLOv5s achieves a 2.5 percentage point increase in average precision. Furthermore, comparative experiments with other enhancement methods demonstrate the effectiveness of the improvement strategies.

## 1. Introduction

With the advancement of drone-related technologies, Unmanned Aerial Vehicles (UAVs), distinguished by their lightweight and swift characteristics, have found extensive applications across diverse domains. Object detection, serving as a pivotal component in the execution of UAV missions, is assuming an increasingly significant role, bearing profound implications for research.

Traditional object detection algorithms primarily rely on classical algorithms in machine learning and computer vision, such as feature-based approaches [1,2], template matching [3], and cascade classifiers [4,5]. These techniques often depend on manually designed features and traditional machine learning algorithms to identify and locate targets. Traditional object detection algorithms typically enhance detection performance through feature fusion and ensemble learning methods. Consequently, traditional approaches to object detection in UAVs generally utilize algorithms predicated on handcrafted features. Shao [6] integrated the Histogram of Oriented Gradients (HOGs) with Support Vector Machine (SVM) for object detection in UAVs. However, in practical applications, traditional object detection algorithms based on handcrafted features exhibit lower stability and demand higher requirements for the detection environment. When changes in lighting conditions or object posture occur, the precision of detection markedly diminishes.

As the realm of deep learning continues to rapidly evolve, object detection algorithms based on deep learning have become a research hotspot in UAV applications. Compared with object detection algorithms based on handcrafted features, those predicated on deep learning boast a wider range of applications, more convenient design, and simpler dataset creation, among other advantages. Object detection methodologies based on deep learning can principally be divided into two categories: the first encompasses two-stage object detection algorithms, exemplified by Fast R-CNN [7] and Faster R-CNN [8], which initially generate candidate regions and subsequently classify and locate objects. These methodologies are characterized by their high detection precision and low omission rates, but they face challenges related to slower detection speeds and demanding computational requirements, rendering them unsuitable for real-time detection. The second category is represented by single-stage object detection algorithms, such as You Only Look Once (YOLO) [9] and Single Shot Multibox Detector (SSD) [10]. These algorithms directly execute the location and category of the target, offering advantages such as swift detection and reduced computational load, albeit at the expense of a relatively lower accuracy. However, in natural scenarios, the substantial distance during drone aerial photography makes it susceptible to environmental factors such as illumination, leading to reduced measurement precision and increased omission rates for small targets. There are two common definitions for small targets. One is common, as defined in the COCO dataset [11], where small targets have a resolution smaller than 32 pixels × 32 pixels. The other definition, based on relative scale, is determined by the target’s proportion to the image, specifically when the target occupies less than 0.01 of the original image ratio. In this paper, objects with a resolution smaller than 32 × 32 pixels or occupying less than 0.01 of the original image ratio are categorized as small target objects. Therefore, the aforementioned mainstream detection algorithms cannot be directly applied to object detection tasks in drone aerial photography scenes.

At present, numerous scholars have embarked on extensive research in the realm of object detection within drone aerial photography scenarios. Liu et al. [12] optimized the darknet Resblock in YOLOv3, while incorporating convolutions in the early layers to increase spatial information. However, as time has passed, the darknet framework appears somewhat antiquated. Luo et al. [13] enhanced detection performance by improving the feature extraction module within the YOLOv5 backbone network and validated the module’s practicality using a substantial dataset. However, their approach exhibited suboptimal results in detecting small objects. Zhou et al. [14], from a data augmentation perspective, devised two data augmentation strategies, namely background replacement and noise addition, to increase the background diversity of the dataset. Although data augmentation improved the detection of small objects to some extent, it merely increased the proportion of small objects in the data, lacking the integration and utilization of semantic information. Wang et al. [15] introduced the Ultra-lightweight Subspace Attention Module (ULSAM) into the network structure, with an emphasis on target features and the attenuation of background features. However, this module primarily incorporated spatial information, neglecting channel information, and resulting in suboptimal small object detection performance, especially in densely occluded scenes. Considering the significant scale discrepancies of objects in drone aerial photography images, Liu et al. [16] proposed a multi-branch parallel feature pyramid network designed to enhance the network’s multi-scale feature extraction capability. However, due to significant disparities in spatial and semantic information among feature maps at different levels, the fusion process easily introduced redundant information and noise, potentially leading to the loss of small object details in different levels. To address the problem of semantic disparities in feature maps at different levels, Wu et al. [17], based on the use of a multi-branch parallel pyramid network, introduced a feature concatenation fusion module. Nevertheless, this method introduced a significant number of additional parameters, which consequently reduced detection speed.

In summary, although existing drone aerial object detection algorithms have improved detection performance to some extent, there are still some shortcomings:

- Inaccurate Localization of Small Objects: The accurate localization of small objects remains a challenge, primarily due to their reduced presence in images in comparison to larger objects. This scarcity poses difficulties in precisely pinpointing their locations.
- Loss of Small Object Feature Information: The downsampling operations commonly applied in detection algorithms can lead to the loss of critical feature information associated with small objects. Recovering these details during the subsequent upsampling stages proves to be a complex endeavor.
- Susceptibility to Confusion Among Small Object Categories: Small objects are particularly susceptible to occlusion and may share similar categories with other objects in their immediate environment. This similarity can result in confusion and misclassification, further complicating the detection process.

Therefore, to address the challenge of detecting small objects in drone scenarios using existing object detection algorithms, this paper endeavors to redesign the network architecture. This redesign involves the integration of multi-scale features, the introduction of attention mechanisms, and the proposal of an enhanced algorithm called SMT-YOLOv5. The primary contributions of this paper’s algorithm are outlined as follows:

- Attention-Based Receptive Field Feature Extraction Module: We introduce an Attention-based receptive field feature extraction module that can be seamlessly integrated into various models. This module efficiently leverages feature information across different scales, capturing a wealth of global contextual cues. Furthermore, it combines spatial and channel attention mechanisms, enhancing the model’s ability to represent crucial information for small objects effectively.
- Detection Layer with Enhanced Small Target Feature Map: We introduce a detection layer featuring a small target feature map sampled at a 4× scale, significantly enhancing our detection capabilities for small objects. Additionally, we incorporate a multi-level feature pyramid structure that facilitates the comprehensive fusion of both local and global information. This fusion markedly improves the accuracy of target detection across various scales. The effective combination of deep and shallow information provides valuable assistance to the network in detecting small objects.
- Dynamic Head: We introduce the DyHead, which cohesively integrates various self-attention mechanisms within the output channels dedicated to scale awareness, spatial awareness, and task awareness. This integration is aimed at enhancing the network’s ability to detect small targets and, consequently, improving the precision of target detection.

The remainder of this paper is structured as follows: Section 2. describes the improved method used in this paper, Section 3. demonstrates the effectiveness of the method in detail through experiments, and Section 4. concludes the paper.

## 2. Proposed Algorithm

### 2.1. Architecture

**Figure 1:**
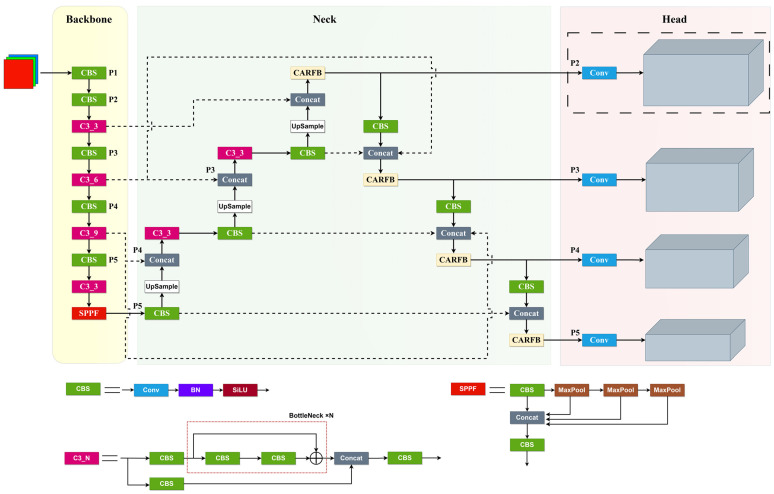
SMT-YOLOV5.

This paper aims to enhance YOLOv5 [18] by addressing specific detection needs in drone aerial images. Focusing on the distinctive detection requirements for drone aerial images, this article has emphasized improvements to three critical components of YOLOv5: feature extraction, feature fusion, and detection heads. It introduces a detection algorithm named SMT-YOLOV5. As illustrated in Figure 1, the algorithm’s framework incorporates a Neck section utilizing the Bi-directional feature pyramid network [19] structure, employing weighted inter-layer feature pyramids. Through bidirectional cross-connections and fast normalization, it effectively integrates features from distinct layers. Furthermore, the introduction of the CARFB module enlarges the receptive field using attention mechanisms, thereby enhancing the detection accuracy for smaller targets. Within the detection head segment, a small target detection layer with 4× downsampling has been introduced. Moreover, the model’s original regression detection head has been substituted with the newly proposed variable detection head, DyHead, to enhance the network’s capability to identify densely packed small targets.

### 2.2. Feature Fusion Network Architecture

#### 2.2.1. Small Object Detection Layer

As depicted in Figure 1, the dashed box denotes the newly added P2 detection branch, specifically designed for detecting extremely small objects. The input to the P2 branch primarily derives from shallow convolutional layers, abundant in information related to shape, position, and size. Nevertheless, deep feature maps might lose significant information following numerous convolution and pooling operations, with features of large objects potentially overshadowing those of smaller objects, resulting in false positives and missed detections. Hence, the incorporation of shallow information through the P2 branch significantly contributes to pinpointing the positions of small objects, thereby improving small object detection. Furthermore, this model utilizes anchor boxes and is particularly sensitive to inaccuracies in box settings. During prediction and regression in the recently introduced P2 detection branch, anchor box sizes are configured based on the dimensions of small objects determined through K-means clustering of the dataset, as outlined in Table 1 for each branch. This enables the P2 branch to address situations where objects are missed due to the use of excessively large anchor boxes for very small objects, effectively reducing false positives and missed detections caused by incorrect box settings.

#### 2.2.2. Improvement for Feature Fusion Path

**Figure 2:**
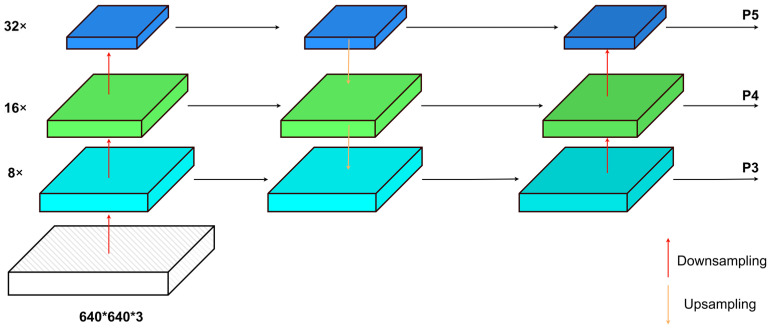
PANet + FPN.

In the domain of small target detection in UAV imagery, a significant challenge involves effectively combining multi-scale features [20]. As shown in Figure 2, The original YOLOv5 algorithm used a cascade architecture comprising the feature pyramid network (FPN) [21] and pyramid attention network (PANet) [22] for feature fusion. This setup established contextual connections to transfer and merge features across different strata. However, variations in feature granularity at different scales have distinct impacts on output characteristics. As deep-level feature maps undergo multiple downsampling iterations, the receptive field expands, leading to increased overlap between different receptive regions. Consequently, the information obtained from features becomes overly fine-grained, unintentionally neglecting the spatial positional data found mainly in shallow-level feature maps. This, in turn, adversely affects the accuracy of small target localization and detection.

The solution to this challenge hinges upon the adept preservation and utilization of information with diverse levels of granularity during the processes of upscaling, downscaling, and tensor concatenation. This is crucial for generating a final feature map with rich spatial and semantic information. Therefore, drawing inspiration from the BiFPN structure, this paper incorporates a skip-connection architecture during the intermediate feature fusion process. Specifically, it engages in feature fusion with the initial input nodes at each output node, facilitating bidirectional cross-scale connections to prevent the loss of spatial positional information for small objects in shallow feature maps. Simultaneously, it expunges the intermediary fusion nodes of the shallowest feature map to maintain the integrity of minor information features. Moreover, it eliminates the intermediary fusion nodes of the deepest feature map due to their minimal contribution to feature fusion.

**Figure 3:**
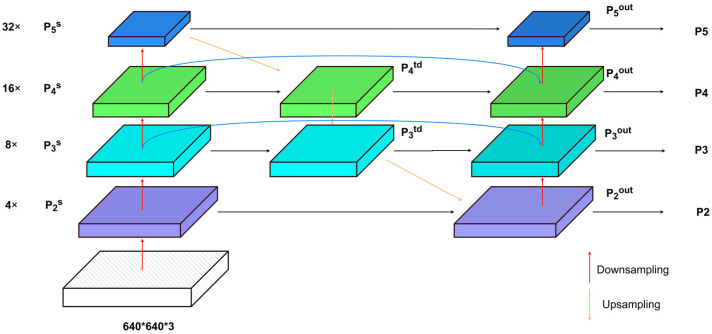
Improved network structure.

As shown in Figure 3, the large-scale feature map $P_{2}^{s}$, resulting from 4× downsampling in the main network, is fused with the top-down processed feature map $P_{3}^{td}$, yielding the $P_{2}^{out}$ feature map. This serves as the input for the P2 layer detection head. The input feature map $P_{3}^{out}$ for the P3 layer detection head employs a skip-connection structure, integrating the features of the 16× downsampled $P_{3}^{s}$ from the main network, the top-down processed feature map $P_{3}^{td}$, and the bottom-up processed feature map $P_{2}^{out}$ (similarly for the P4 layer detection head). The input feature map $P_{5}^{out}$ for the P5 layer detection head is a fusion of the 32× downsampled feature map $P_{5}^{s}$ from the main network and the top-down and bottom-up fused feature map $P_{4}^{out}$ from the feature fusion network. The fusion of feature layers at different resolutions is accomplished through a weighted fusion approach. The fusion process employs fast normalized feature fusion.

Fast normalized feature fusion deviates from conventional fusion techniques. Typically, fusing features of different resolutions involves aligning them to the same resolution and summing them. Fast normalized feature fusion, on the other hand, takes into account that different input features have different resolutions and unequal contributions to the output features. Therefore, it adds additional weights to each input to let the network learn the importance of each input feature. The weighted fusion method combines features of different resolutions, assigns a weight to each input, and allows the network to adjust the fusion weights for different inputs. The formula for fast normalized feature fusion is as follows:

(1) $$O=\sum_{i}\frac{w_{i}}{\varepsilon +\sum_{j}w_{j}}\cdot I_{i}$$

where $I_{i}$ is the input feature, $w_{i}$ is the learnable weight for each input feature, subscripts *i* and *j* are the layer indices, and *ε* = 0.0001 is a small additional value to maintain numerical stability.

The realization of feature fusion between the upper and lower layers in the aforementioned process is as follows (excluding the fusion of $P_{2}^{out}$ and $P_{4}^{td}$):

(2) Pitd=Conv[w1·Pis+w2·Re size(Pi+1td)w1+w2+ε]

(3) Piout=Conv[w1′·Pis+w2′·Pitd+w3′·Re size(Pi−1out)w1′+w2′+w3′+ε]

In Formulas (2) and (3), $P_{i}^{s}$ represents the input features of the i-th layer; $P_{i}^{td}$ is the intermediate feature of the top-down path at layer i; $P_{i}^{out}$ is the output feature of the bottom-up path at layer i; $w_{1}$ and $w_{2}$ are the learned weights associated with the input features; $w_{1}^{\prime }$, $w_{2}^{\prime }$, and $w_{3}^{\prime }$ are the updated learned weights following the preceding layer’s computation; Conv() corresponds to convolution operation; and Resize is the sampling operation.

The feature fusion network employed in this article boasts several advantages over the original YOLOv5 feature fusion network connectivity:

- It enhances feature propagation efficiency. Due to the limited contribution of nodes lacking feature fusion to feature network propagation computations, the intermediate nodes of P2 and P5 are excised, yielding a streamlined bidirectional network. This refinement notably augments network propagation efficiency.
- It effectively amalgamates features of varying resolutions, heightening the sensitivity of output features to small object detection. Diverging from the Path Aggregation Network with only one top-down feature path and one bottom-up feature path, the BiFPN interlaces features in both top-down and bottom-up directions. Moreover, through normalization operations, it endows each input with varying significance for the detection network, thereby elevating the weightage of small targets. Consequently, this bolsters the network’s expressive prowess and feature extraction efficacy.

### 2.3. Attention-Based Receptive Field Feature Extraction Module

In common drone aerial scenarios, the scale of objects within images exhibits variability. As the network structure deepens and undergoes multiple convolution operations, small targets tend to lose a substantial amount of crucial feature information, rendering them challenging to detect and identify within high-level feature maps. Therefore, acquiring feature information of various scale sizes is crucial to enhance the reliability of small target detection. While Liu et al.’s [23] Receptive Fields Block (RFB) can capture image features across various scales and attain different receptive field sizes, the extracted feature information is extensive and lacks a focus on critical details, resulting in a less than ideal detection performance for small targets.

To address this issue, this paper introduces a Receptive Fields Feature Extraction Module, named Combine Attention and Receptive Fields Block, which integrates both channel and spatial attention mechanisms. This module takes into account the feature variations between different receptive field channels to enhance the expression of feature information. The Convolutional Block Attention Module (CBAM) [24] is an attention module employed to improve convolutional neural networks by combining channel and spatial attention. It assists in capturing the significance of various channels within the input feature map and identifying the importance of different locations on the feature map. The introduction of the CBAM module not only allows RFB to cover a larger area for capturing rich feature information but also employs attention mechanisms to extract critical features from the abundant feature information. Consequently, it enhances the model’s ability to detect multi-scale and dense small targets in complex backgrounds.

**Figure 4:**
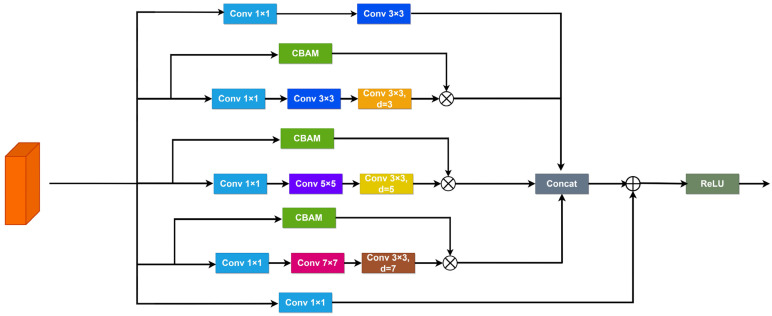
Architecture of CARFB.

Illustrated in Figure 4, the CARFB structure is composed of five parallel branches. The first branch consists of a 1 × 1 and a 3 × 3 convolutional layer, aimed at extracting information from the input feature map without dilation. The three central branches utilize dilation rates of three, five, and seven, each integrated with a CBAM module to gather comprehensive feature information while emphasizing essential details. Subsequently, the last branch includes only a 1 × 1 convolutional layer to reduce the number of channels. The feature maps extracted from the first four branches are concatenated and added to the original input feature information from the fifth branch, forming a residual structure.

The computational process unfolds as follows:

(4) $$F_{1}=f_{r=1}^{3\times 3}(f^{1\times 1}(I))$$

(5) $$F_{2}=f_{r=3}^{3\times 3}(f^{3\times 3}(f^{1\times 1}(I)))$$

(6) $$F_{3}=f_{r=5}^{3\times 3}(f^{3\times 3}(f^{3\times 3}(f^{1\times 1}(I))))$$

(7) $$F_{4}=f_{r=7}^{3\times 3}(f^{3\times 3}(f^{3\times 3}(f^{3\times 3}(f^{1\times 1}(I)))))$$

(8) $$OUT=relu(f^{1\times 1}(Concat(F1,F2,F3,F4)+F5))$$

where I is the input feature map, and O represents the output feature map. For computational simplification, the actual code utilizes two 3 × 3 convolutions in place of the 5 × 5 convolution illustrated in the diagram, and three 3 × 3 convolutions replace the 7 × 7 convolution.

### 2.4. Dynamic Head

The intricacy inherent in the localization and classification aspects of object detection fundamentally arises from the inherent conflict between translational invariance and image scale invariance within convolutional neural networks. This dilemma is exacerbated by the presence of multiple objects in real-world images, each possessing distinct proportions and sizes. Furthermore, these objects might exhibit markedly different shapes and positions when viewed from diverse perspectives. To surmount this challenge, the head segment of object detection ought to possess a certain degree of spatial perceptiveness.

**Figure 5:**
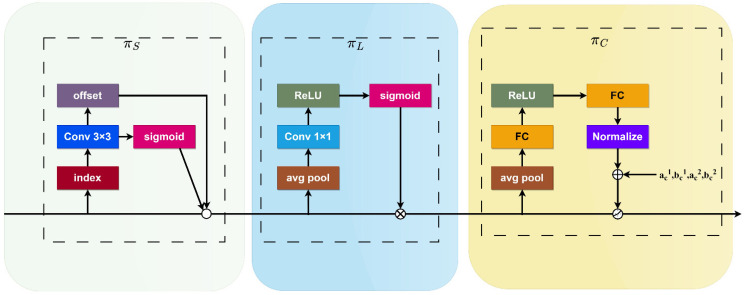
Architecture of DyHead.

This study introduces the dynamic detection head [25], supplanting the previously utilized detection head. This detection head seamlessly integrates various self-attention mechanisms to adapt to the diversity of feature level importance between scale-aware feature hierarchies and the spatial awareness of spatial positions. It enables adaptability to input data. The specific structure is depicted in Figure 5.

For a given feature pyramid, let us denote the four-dimensional tensor as *F*∈$R^{L\times S\times C}$, where *L* represents the number of layers in the pyramid, *S* = *H* × *W*, and *H*, *W*, and *C*, respectively, stand for the height, width, and number of channels of the feature.

The self-attention expression is as follows:

(9) W(F)=π(F)·F

where *π* (·) is the attention function, implemented through a fully connected layer. However, directly learning the attention function across all dimensions can be computationally intensive. Therefore, this article transforms the attention function into three consecutive attentions, each focusing on a specific direction. This decomposition helps handle the relationship between features at different hierarchies and object scales, improving the representation learning of different hierarchical features and aiding in enhancing scale awareness in object detection. Thus, based on the semantic importance at different scales, the proposed dynamic fusion feature equation is as follows:

(10) $$W(F)=\pi_{C}(\pi_{L}(\pi_{S}(F)\cdot F)\cdot F)\cdot F$$

In Formula (11), the symbols are defined as follows.

Spatial-aware Attention $\pi_{S}(F)$: Sparse spatial attention is obtained through deformable convolution to adaptively sample spatial locations via additional self-learned offsets. This approach not only applies attention to each spatial location but also adaptively aggregates multiple feature layers to learn more distinctive representations.

(11) $$\pi_{S}(F)\cdot F=\frac{1}{L}\sum_{l=1}^{L}\sum_{k=1}^{K}\omega_{l,k}\cdot F(l;p_{k}+\Delta p_{k};c)\cdot \Delta m_{k}$$

where *K* is the number of sparsely sampled positions, $p_{k}+∆p_{k}$ represents the self-learned spatial offset, and self-learning $∆p_{k}$ is used to focus on distinctive regions.

Scale-aware Attention $\pi_{L}(F)$: This dynamically fuses features based on the semantic importance at different scales.

(12) $$\pi_{L}(F)\cdot F=\sigma (f(\frac{1}{SC}\sum_{S,C}F))\cdot F$$

where the function is approximated as a linear function using a 1 × 1 convolutional layer, and *σ(x)* represents a Sigmoid function.

Task-aware Attention $\pi_{C}(F)$: This utilizes the DYReLU-b dynamic ReLU activation function. First, global average pooling is performed on L × S dimensions to reduce dimensionality. Then, it is processed through two fully connected layers and a normalization layer, and finally, the output is normalized to [−1, 1] using a Sigmoid function.

## 3. Experimental Results and Analysis

### 3.1. Dataset and Experimental Environment

#### 3.1.1. Dataset

**Figure 6:**
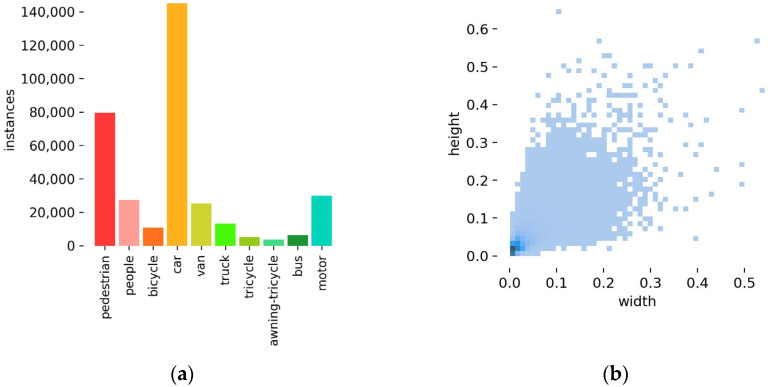
The visualized results of the attributes of the Visdrone dataset used in this paper. (**a**) The categories of dataset. (**b**) The ratio of the height to the width of the bounding box to the original image.

This paper used the VisDrone2021 [26] dataset for the training and performance evaluation of SMT-YOLOv5. The VisDrone2021 dataset is a publicly available object detection dataset based on drone vision collected by the AISKYEYE team at Tianjin University. It includes images captured by different models of drones at various locations and heights under different scenes, weather conditions, and lighting conditions. Consequently, the images contain numerous high-density small objects, referring to densely distributed and abundant small targets within the images. The dataset comprises a total of 8599 images, divided into a training set (6471 images), a validation set (548 images), and a test set (1580 images). The VisDrone dataset consists of 10 categories with approximately 540,000 annotations. The distribution of object categories for individual instances is shown in Figure 6a. Cars and pedestrians make up the majority of the objects, while tricycles, buses, etc., have a smaller presence, resulting in an imbalanced category distribution. Figure 6b illustrates the proportions of objects in different size ranges within the images, with most objects being small and only spanning a few tens of pixels. The category imbalance and a substantial number of small objects effectively test the algorithm’s ability to detect and recognize small objects.

#### 3.1.2. Experimental Parameter Configuration

The experimentation environment for this research is based on the Windows 11 operating system. The training and inference processes of the model were conducted on an RTX 3060Ti GPU. The deep learning framework utilized is PyTorch 1.13, with a CUDA version of 11.7. The experimental parameters include a batch size of 8, training for 200 epochs, an input size of 640, and a learning rate of 0.01. The optimization method employed is Stochastic Gradient Descent (SGD) with momentum, with a momentum parameter of 0.937 and a weight decay coefficient of 0.0005. The training commences from scratch without leveraging any pre-trained weights.

### 3.2. Experimental Evaluation Metrics

To assess the detection performance of our proposed enhanced model, we employ precision, recall, mAP0.5, mAP0.5:0.95, $AP_{s}$, $AP_{m}$, $AP_{l}$, number of model parameters, and model size as evaluation metrics, and their calculation formulas are detailed in Table 2.

In Table 2, TP represents the true positives, which are actual positives correctly classified by the classifier. FP stands for false positives, indicating actual negatives incorrectly classified as positives. FN represents false negatives, representing actual positives incorrectly classified as negatives. TN denotes true negatives, which are actual negatives correctly classified as negatives by the classifier. In general, as R increases, P tends to decrease. AP, calculated as the area enclosed by the curve when precision is plotted against recall, is a key metric in object detection. In this context, a higher AP indicates better classification performance. mAP represents the mean average precision, calculated as the average of the AP values for all classes. It is commonly used to measure the overall performance of an algorithm. In the mAP calculation formula, $AP_{i}$ represents the AP value for the class with index i, and N represents the number of classes in the training dataset (in this paper, N is 10). mAP0.5 denotes the average precision when the detection model’s Intersection over Union (IoU) is set to 0.5. mAP0.5:0.95 represents the average precision when the detection model’s IoU is set in the range of 0.5 to 0.95, with increments of 0.05. $AP_{s}$, $AP_{m}$, and $AP_{l}$ are selected as the evaluation indexes of small, medium, and large targets, respectively.

### 3.3. Experiment Results

#### 3.3.1. Comparative Experiment of Small Object Detection Head

To verify the advantages of introducing a small target detection head, we conducted comparative experiments under consistent training conditions, both before and after the addition of the small target detection head. The experimental results, as shown in Table 3, indicate that when adding the 4× downsampled small object detection head, mAP@0.5 improves by 3% compared to YOLOv5s. When small objects undergo numerous convolution and pooling operations, the feature maps can become significantly compressed, leading to feature loss. Adding detection layers helps mitigate this feature loss issue, resulting in a significant enhancement in detection performance for small objects, despite the increase in computational cost.

#### 3.3.2. Comparative Experiment of CARFB

This study aims to validate the effectiveness of the proposed CARFB module for improving the accuracy of small object detection. We added the CARFB module to the YOLOv5-xs model, which has an additional small object detection head, and compared the detection accuracy before and after the addition. A comparison of the model performance is shown in Table 4: The YOLOv5-xs-RFB model’s mAP@0.5 increased by only 0.9% compared to YOLOv5-xs, while mAP@0.5 increased by 1.4%. In addition, precision increased by 1%, and recall increased by 1.4%. The improvement in average precision is primarily due to the introduction of multi-scale receptive fields during the feature extraction process, enabling the network to effectively extract features of objects of different sizes. The experimental results demonstrate that the CARFB module can effectively enhance feature extraction attention for small objects during the feature extraction process.

#### 3.3.3. Comparative Experiment of BiFPN

The purpose of this section is to validate the detection performance of the proposed BiFPN feature fusion structure. Using the YOLOv5-xs model with an added module for small object detection as the baseline, we conducted feature fusion in two different ways: PANet + FPN and BIFPN, followed by a comparative analysis. The experimental results are presented in Table 5.

From Table 5, it is evident that adopting the BIFPN feature fusion approach, as opposed to the originally used PANet + FPN, led to a 0.6 increase in GFLOPS. However, it also resulted in a 1.3% improvement in mAP@0.5, a 0.9% increase in mAP@0.5:0.95, a 1.5% boost in recall, and a marginal 0.2% increase in precision. Therefore, despite a slight increase in model complexity, the BiFPN feature fusion approach successfully enhances the detection accuracy for small objects.

#### 3.3.4. Comparative Experiment of DyHead

By controlling the stacking of different numbers of DyHead blocks, we evaluate their impact on model performance and computational cost. The experimental baseline model utilizes YOLOv5s-xs-BiFPN and adds 1, 2, and 6 DyHead blocks to the baseline model. The model with zero added blocks serves as the baseline. The experimental results are presented in Table 6.

From Table 6, it can be observed that the accuracy increases with the increasing number of stacked DyHead blocks, albeit with a slight increase in computational cost and parameter count. This paper takes into account both the accuracy and complexity of the algorithm. Therefore, it chooses to integrate four DyHead blocks into the algorithm, achieving an mAP@0.5 of 43.4%, with GFLOPS at 29.2.

### 3.4. Experimental Results of SMT-YOLOV5

**Figure 7:**
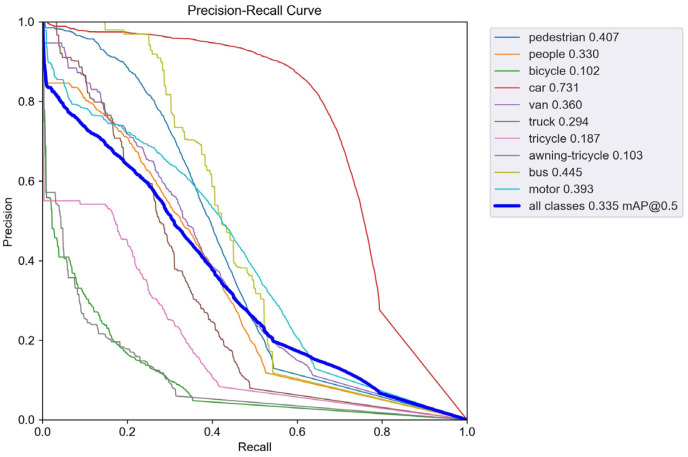
PR-curve for YOLOv5s.

**Figure 8:**
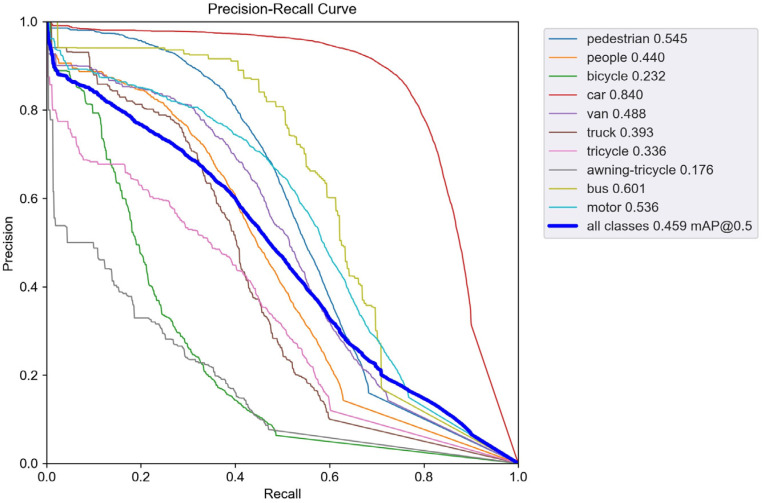
PR-curve for SMT-YOLOv5s.

To validate the performance improvement in the enhanced model, we conducted comparative experiments between the improved model and the baseline YOLOv5s. Figure 7 shows the precision–recall (PR) curve performance of YOLOv5s on the VisDrone dataset, while Figure 8 displays the PR curve performance of SMT-YOLOv5s on the same dataset. The experimental results indicate that the improved SMT-YOLOv5s algorithm exhibits various degrees of improvement in the AP values for all classes. The AP for categories such as pedestrians, cars, trucks, buses, people, vans, and buses has increased by more than 10%. The highest improvement is observed in the tricycle category, with an increase of 14.9%. Even the most challenging to detect, the awning-tricycle, shows a 7.3% improvement. This suggests that the improved model effectively enhances the detection accuracy for small objects and overall detection performance. The data in Table 7 indicate that SMT-YOLOv5 outperforms YOLOv5 significantly across large, medium, and small targets, showing improvements of 6.9%, 9.5%, and 7.7%, respectively. This suggests that the enhanced model effectively enhances the accuracy of both small object detection and overall detection performance. Furthermore, from the figures, it can be observed that the SMT-YOLOv5s curve is smoother, more continuous, and more stable. PR curves are sensitive to data imbalance, and changes in the positive-to-negative sample ratio can cause significant variations in the curve. The improved algorithm exhibits higher accuracy, resulting in a more balanced positive-to-negative sample ratio and enhanced robustness. SMT-YOLOv5s demonstrates a more comprehensive and robust detection capability.

### 3.5. Analysis of Detection Performance

**Figure 9:**
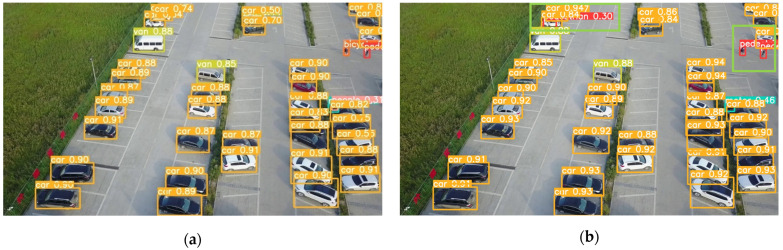
YOLOv5s vs. SMT-YOLOv5: dense distribution detection. (**a**) result of YOLOv5; (**b**) result of SMT-YOLOv5.

**Figure 10:**
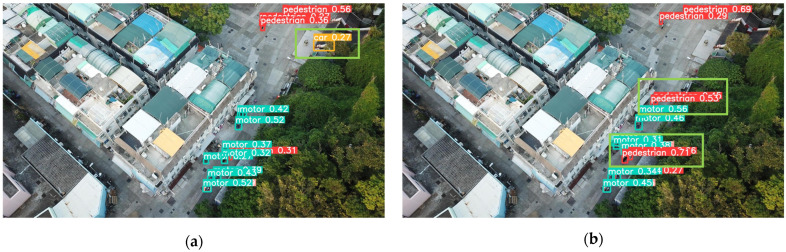
YOLOv5s vs. SMT-YOLOv5: complex background detection. (**a**) result of YOLOv5; (**b**) result of SMT-YOLOv5.

**Figure 11:**
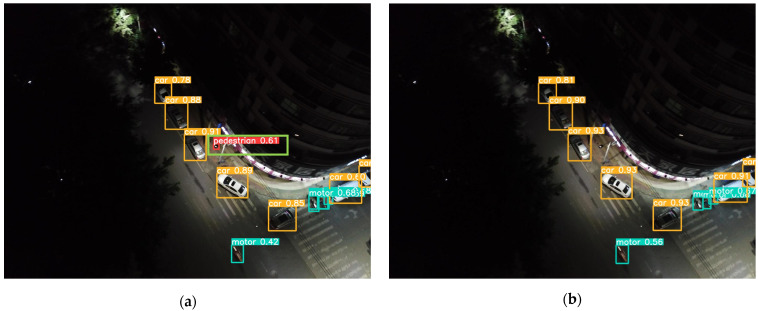
YOLOv5s vs. SMT-YOLOv5: low illumination detection. (**a**) result of YOLOv5; (**b**) result of SMT-YOLOv5.

**Figure 12:**
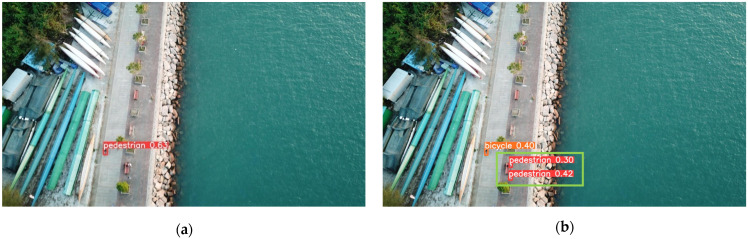
YOLOv5s vs. SMT-YOLOv5: minuscule target detection. (**a**) result of YOLOv5; (**b**) result of SMT-YOLOv5.

To validate the detection performance of the SMT-YOLOv5 algorithm in real-world scenarios, this study explored its capabilities in specific scenarios. One image from each of the densely populated areas, complex backgrounds, images with small objects, and low-light conditions was selected. YOLOv5 and SMT-YOLOv5 were tested on these images, and the comparative results are shown in Figure 9, Figure 10, Figure 11 and Figure 12. The left side of the figure displays the detection results of YOLOv5, while the right side shows the detection results of SMT-YOLOv5.

Through comparison, it is evident that in scenarios such as parking lots, residential buildings, night-time streets, and roads near water, the performance of SMT-YOLOv5s is markedly superior to YOLOv5s. It demonstrates precise target detection in dense, long-distance, and corner areas, exhibiting greater accuracy in target delineation and recognition. In Figure 9a, YOLOv5s fails to detect pedestrians alighting from vehicles in a crowded parking lot. Moreover, due to the distant shooting distance, the pedestrian walking on the ground is erroneously classified as a bicycle. Conversely, as shown in Figure 9b, SMT-YOLOv5s accurately identifies the pedestrian alighting in the crowded parking lot without misclassifying them as a bicycle. In Figure 10a, YOLOv5s incorrectly identifies the white eaves in the upper right corner of the image as a parked car, possibly due to tree obstruction or environmental light influence. As a small portion of the house’s eaves is obscured, pedestrians under the eaves are not detected. On the contrary, as shown in Figure 10b, SMT-YOLOv5s does not produce false detections and accurately detects pedestrians under the eaves. In Figure 11a, under night-time and low-light conditions, YOLOv5s incorrectly identifies the lamppost next to the white car as a pedestrian. In contrast, as evident in Figure 11b, under the same lighting conditions, SMT-YOLOv5s does not encounter such issues. In Figure 12a, YOLOv5s incorrectly identifies the moving bicycle as a pedestrian and fails to accurately detect pedestrians on seats. However, as shown in Figure 12b, the proposed SMT-YOLOv5s algorithm comprehensively learns features, accurately identifying people sitting on seats and pedestrians walking on the road without false detections. Through the comparison of the above images, it is observed that SMT-YOLOV5S accurately detects targets missed by YOLOv5s and is more precise in identifying individual target points than YOLOv5s.

**Figure 13:**
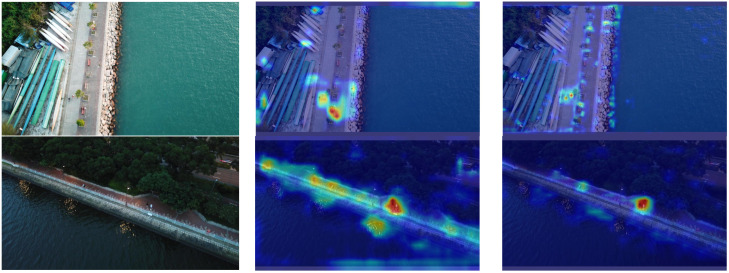

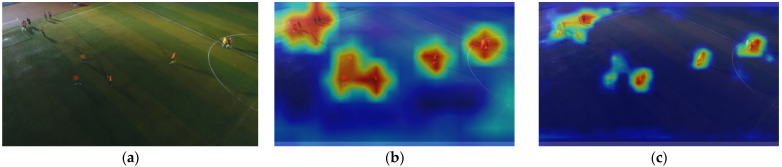
YOLOv5s vs. SMT-YOLOv5: heatmap comparison for tiny objects. (**a**) Original image, (**b**) YOLOv5s result, (**c**) SMT-YOLOv5s result.

To further validate and analyze the improvements in the SMT-YOLOv5 model in small object detection, this paper employs the Gradient-weighted Class Activation Mapping (Grad-CAM) [27] method to analyze and compare the heat maps of the proposed model and YOLOv5. Grad-CAM, a gradient-based localization method, serves to visualize deep neural networks and showcase the features acquired through convolutional networks. The method involves computing the weight of each feature map, deriving the global average of gradients, and executing backward propagation to obtain gradient values. Such an approach facilitates the analysis of the network’s focal regions concerning a particular class, allowing for a retrospective assessment of whether the network has accurately acquired specific features or information. More specifically, within the heatmap, deeper shades of red signify a greater contribution of the region to the final prediction, signifying the network’s heightened attention to this portion of the image. Conversely, deeper shades of blue suggest a diminished contribution to the final prediction, implying that the network considers this information to be redundant. The experimental results are shown in Figure 13.

From Figure 13, we can infer that YOLOv5 exhibits limited attention to small objects, primarily focusing on areas unrelated to the target, such as the adjacent road and ground regions. Additionally, it tends to overlook the existence of small objects and is not very sensitive to distant objects. In contrast, the model proposed in this paper demonstrates effective noise suppression in the background and places greater emphasis on small targets. This model focuses more on the center point of the target, resulting in more precise predictions of bounding boxes, ultimately enhancing the overall detection performance of the model.

### 3.6. Ablation Experiments

To verify the effects of each improvement strategy proposed in this paper, we conducted a series of ablation experiments on the basis of YOLOv5 utilizing the VisDrone 2021 dataset, and the experimental results are shown in Table 5. Initially, the experiment incorporated the newly added detection branch P2 based on the original YOLOv5s. Subsequently, the structure in the Neck was replaced with the BiFPN feature pyramid network structure. Following this, the CARFB receptive field feature extraction module, based on the attention mechanism, is added. Ultimately, the coupled detection head is substituted with the dynamic detection head DyHead. To ensure the fairness of the comparison, different experiments only incrementally added the corresponding modules, without altering the optimization methods or hyperparameters.

According to the results of the ablation experiments in Table 8, the model improvements in this study have significantly enhanced the accuracy of small object detection. Firstly, the comparative experimental results show that the newly added detection branch P2 has a significant improvement effect on the mAP@0.5 metric, reaching three percentage points. This indicates that the newly added detection branch is very effective for small object detection, and it also suggests that setting the anchor boxes of the newly added P2 detection branch to the size of small objects can greatly reduce missed detections caused by setting anchor boxes too large. Secondly, the BiFPN structure, with only a 0.6 increase in GFLOPS, improved mAP@0.5 by 1.3 percentage points. This suggests that through multi-level information fusion and combining shallow shape and size information, the detection performance of small objects can be enhanced. In addition, the CARFB receptive field feature extraction module has achieved a 1.7 percentage point improvement in the mAP@0.5 metric, indicating that introducing a feature fusion module with an attention mechanism can enhance focus on small objects, thereby improving detection performance. Finally, replacing the detection head with a stack of four DyHead detection heads has the most pronounced effect, with mAP@50 improving by 6.8 percentage points and mAP@0.5:0.95 improving by 4.9 percentage points. Although the complexity of the model also increased, with GFLOPS increasing by 10.1 to reach 32.8, it remains within an acceptable range. This indicates that introducing attention mechanisms in the dimensions of detection head scale, space, and task can improve the accuracy of object detection. Furthermore, the P and R values in the table consistently improve after each modification to the model, indicating that each added module has a positive impact on the detection performance of small objects.

### 3.7. Comparison of Different YOLO Versions

To authenticate the performance of the SMT-YOLOv5 algorithm, this study conducted comparative experiments with other versions of the YOLO algorithm, the results of which are displayed in Table 9.

As can be discerned from the experimental results in Table 9, SMT-YOLOv5 boasts the highest detection precision and the most optimal comprehensive detection performance. SMT-YOLOv5 not only exhibits superiority over YOLOv5s but also holds advantages when compared with YOLOv3 [28], YOLOv5l, YOLOv7 [29], and YOLOv8s, and models proposed by other scholars.

The mAP@0.5 of SMT-YOLOv5s is 7.6 percentage points, 12.4 percentage points, and 6.6 percentage points higher than YOLOv3, YOLOv5s, and YOLOv8s, respectively. Although early YOLO series algorithms (such as YOLOv3) can achieve an mAP@0.5 value of 38.3, their complex structure and large number of parameters make them unsuitable for deployment on drone platforms. The model sizes of YOLOv5s and YOLOv8s are smaller and have fewer parameters, but both models use a three-scale model structure, which cannot meet the detection needs of high-ratio small objects. Furthermore, both YOLOv8s and YOLOv5s adopt the original model’s depth multiple of 0.33 and width multiple of 0.5. However, YOLOv8s with a GFLOPS of 28.8 and YOLOv5s with a GFLOPS of 15.8 reveal a substantial discrepancy in computational complexity under equivalent model sizes. Recognizing the need for lightweight solutions, we opted for YOLOv8n with a GFLOPS of 8.1 for experimentation. Trained under identical model conditions, SMT-YOLOv8n achieved an mAP@0.5 of 43.4, which is still 2.5 percentage points lower than SMT-YOLOv5s. Therefore, under comparable GFLOPS magnitudes, their detection accuracy falls short of the SMT-YOLOv5 model proposed in this study. Therefore, at the same size, their detection precision is lower than the SMT-YOLOv5 model proposed in this study. It is worth mentioning that compared with the models proposed by other scholars (such as KPE-YOLOv5s [30], UN-YOLOV5s [31], and FE-YOLOv5s [32]), SMT-YOLOv5s has a superior performance in terms of mAP@50, mAP@0.5:0.95, and GFLOPS values. KPE-YOLOv5s redesigns the size of the anchor box using the K-Means++ clustering algorithm and introduces the SE attention module, but the network feature fusion part lacks optimization, resulting in relatively poor small object detection precision. UN-YOLOv5s proposes a multi-scale feature fusion path and introduces a new convolutional SimAM residual module, but this increases the complexity of computation and training. FE-YOLOv5 design integrates a Space-Aware Module (SAM) to filter spatial information and enhance the robustness of the features. However, the improvement effect is not significant.

In summary of the aforementioned comparative experiments, our proposed multi-scale feature fusion network achieves four-scale detection. This small object detection structure has certain advantages over the comparative experimental models involved; hence, our detection results are superior to other models. In addition, we introduced multiple receptive field feature extraction modules and dynamic detection heads in the baseline model, optimizing the model’s feature extraction capability and noise suppression ability. The improvement strategies we introduced take into account resource consumption, thereby achieving better detection results.

## 4. Conclusions

Addressing the deficiencies of existing object detectors in small object detection, such as false detection and omission, this study proposes an enhanced detection model, SMT-YOLOv5, predicated on YOLOv5. Firstly, to address the difficulty of detecting small objects in drone imagery, we add a detection layer in the feature fusion network to enhance the ability to capture small objects, while employing a weighted bi-directional feature pyramid network capable of effectively integrating information from different receptive fields. This approach resolves the lack of sufficient high-level semantic information and effective fusion between multi-scale receptive fields, thereby improving the detection accuracy of small objects. Subsequently, a receptive field feature extraction module, CARFB, based on the attention mechanism, is introduced to expand the receptive field of the feature map and reduce feature information loss. Building upon this, a dynamic object detection head, DyHead, is incorporated to enhance perception in three dimensions, space, scale, and task, addressing the issue of objects presenting drastically different shapes and positions under different natural viewing angles, and improving the detection accuracy of occluded high-density small objects. Finally, experimental validation on the VisDrone2021 dataset attests to the remarkable enhancement achieved by SMT-YOLOv5 in the realm of target detection accuracy. Each refinement strategy augments mean precision. Ultimately, relative to the original methodology, SMT-YOLOv5s exhibits an elevation of 12.4 percentage points in mean precision. Furthermore, in the detection of large, medium, and small targets, improvements of 6.9%, 9.5%, and 7.7%, respectively, are observed compared to the original approach. Similarly, the application of identical enhancement strategies to the computationally less intricate YOLOv8n yields SMT-YOLOv8n, presenting a complexity akin to that of SMT-YOLOv5s. The results manifest that, in comparison to SMT-YOLOv8n, SMT-YOLOv5s demonstrates a 2.5 percentage point increase in mean precision. Additionally, in comparative experiments with alternative enhancement methodologies such as KPE-YOLOv5s, UN-YOLOv5s, and FE-YOLOv5s, our proposed approach showcases increments of 6.7 percentage points, 5.4 percentage points, and 8.9 percentage points in mAP@0.5, respectively, affirming the efficacy of our refinement strategies. Naturally, what brings us delight is that the same approach yields commendable results on YOLOv8n, providing a guiding direction for our subsequent enhancements.

## Figures and Tables

**Table 1:** Anchor settings for each detection branch.

Detection Branch	Anchor Frame Configuration
P2	(3,4), (6,5), (4,8), (11,6)
P3	(6,12), (11,11), (10,20), (20,10)
P4	(17,18), (31,16), (17,32), (30,39)
P5	(46,26), (45,64), (81,54), (94,84)

**Table 2:** Evaluation metrics and their definitions.

Evaluation Metrics	Metric Definitions
Precision (P)	$P=\frac{TP}{TP+FP}$
Recall (R)	$R=\frac{TP}{TP+FN}$
Average Precision (AP)	$AP=\int_{0}^{1}P(R)dR$
Mean Average Precision (mAP)	$mAP=\frac{\sum_{i=1}^{k}AP_{i}}{k}$
$AP_{s}$	AP for small objects area $<32^{2}$
$AP_{m}$	AP for medium objects $32^{2}<$ area $<96^{2}$
$AP_{l}$	AP for large objects area $>32^{2}$

**Table 3:** Comparison of small object detection results.

Models	P(%)	R(%)	mAP@0.5 (%)	mAP@0.5:0.95 (%)	GFLOPs
YOLOv5s	44.5	34.2	33.5	17.4	15.8
YOLOv5s-xs	47.2	36.7	36.5	20.1	18.8

**Table 4:** Comparison of CARFB module results.

Models	P (%)	R (%)	mAP@0.5 (%)	mAP@0.5:0.95 (%)	GFLOPs
YOLOv5s-xs	47.2	36.7	36.5	20.1	18.8
YOLOv5s-xs-CARFB	48.2	37.5	37.9	21	20.8

**Table 5:** Comparison of feature fusion results with BiFPN.

Models	P(%)	R(%)	mAP@0.5 (%)	mAP@0.5:0.95 (%)	GFLOPs
YOLOv5s-xs	47.2	36.7	36.5	20.1	18.8
YOLOv5s-xs-BiFPN	47.4	38.2	37.8	21	19.4

**Table 6:** The impact of the number of DyHead layers on model performance.

The Quantity of DyHead	P (%)	R (%)	mAP@0.5 (%)	mAP@0.5:0.95 (%)	GFLOPs
0	47.4	38.2	37.8	21	19.4
1	51	39.7	40.6	23	22.3
2	53.3	39.9	41.5	23.8	24.8
4	53.4	42.1	43.4	24.9	29.2
6	54.2	42.8	44.5	25.6	33.4

**Table 7:** Comparison of performance among large, medium, and small targets.

Models	$AP_{s}$ (%)	$AP_{m}$ (%)	$AP_{l}$ (%)
YOLOv5s	10.1	24.7	29.4
SMT-YOLOv5s	17.8	34.2	36.3

**Table 8:** Comparison of ablation experiment metrics.

Methods	P (%)	R (%)	mAP@0.5 (%)	mAP@0.5:0.95 (%)	GFLOPs
YOLOv5s	44.5	34.2	33.5	17.4	15.8
+P2	47.2	36.7	36.5	20.1	18.8
+BiFPN	47.4	38.2	37.8	21	19.4
+CARFB	50.5	38.8	39.1	21.8	22.7
+Dyhead	56.1	43.7	45.9	26.7	32.8

**Table 9:** Compared with other YOLO series algorithms.

Methods	P (%)	R (%)	mAP@0.5 (%)	mAP@0.5:0.95 (%)	GFLOPs
YOLOv3	49.2	38.3	38.3	23.3	154.7
YOLOv5s	44.5	34.2	33.5	17.4	15.8
YOLOv5l	49.9	38.2	38.3	21.5	107.8
YOLOv8s	50.9	45.6	39.3	23.5	28.8
YOLOv8n	40.7	31.6	30.5	17.4	8.1
YOLOv7	51.4	42.1	39.9	21.6	103.5
KPE-YOLOv5s	52.5	39.1	39.2	/	/
UN-YOLOv5s	48.9	40.4	40.5	22.5	37.4
FE-YOLOv5s	/	/	37	20.7	31
SMT-YOLOv5s	56.1	43.7	45.9	26.7	32.8
SMT-YOLOv8n	54	41.1	43.4	26.4	29.8

## Data Availability

The data that support the study are available from the corresponding author upon reasonable request.
